# Multimodal In-Vehicle Hypoglycemia Warning for Drivers With Type 1 Diabetes: Design and Evaluation in Simulated and Real-World Driving

**DOI:** 10.2196/46967

**Published:** 2024-04-18

**Authors:** Caterina Bérubé, Martin Maritsch, Vera Franziska Lehmann, Mathias Kraus, Stefan Feuerriegel, Thomas Züger, Felix Wortmann, Christoph Stettler, Elgar Fleisch, A Baki Kocaballi, Tobias Kowatsch

**Affiliations:** 1 Centre for Digital Health Interventions Department of Management, Technology and Economics ETH Zurich Zurich Switzerland; 2 Department of Diabetes, Endocrinology, Nutritional Medicine and Metabolism Bern University Hospital University of Bern Bern Switzerland; 3 School of Business, Economics and Society Friedrich-Alexander-Universit¨at Erlangen-Nürnberg Nürnberg Germany; 4 LMU Munich School of Management LMU Munich Munich Germany; 5 Department of Endocrinology and Metabolic Diseases Kantonsspital Olten Olten Switzerland; 6 Institute of Technology Management University of St.Gallen St Gallen Switzerland; 7 School of Computer Science University of Technology Sydney Sydney Australia; 8 Institute for Implementation Science in Health Care University of Zurich Zurich Switzerland

**Keywords:** digital health, voice assistant, ambient lighting, in-vehicle technology, health state, diabetes, hypoglycemia, warning, emotional reaction, technology acceptance, mobile phone, diabetes, implementation

## Abstract

**Background:**

Hypoglycemia threatens cognitive function and driving safety. Previous research investigated in-vehicle voice assistants as hypoglycemia warnings. However, they could startle drivers. To address this, we combine voice warnings with ambient LEDs.

**Objective:**

The study assesses the effect of in-vehicle multimodal warning on emotional reaction and technology acceptance among drivers with type 1 diabetes.

**Methods:**

Two studies were conducted, one in simulated driving and the other in real-world driving. A quasi-experimental design included 2 independent variables (blood glucose phase and warning modality) and 1 main dependent variable (emotional reaction). Blood glucose was manipulated via intravenous catheters, and warning modality was manipulated by combining a tablet voice warning app and LEDs. Emotional reaction was measured physiologically via skin conductance response and subjectively with the Affective Slider and tested with a mixed-effect linear model. Secondary outcomes included self-reported technology acceptance. Participants were recruited from Bern University Hospital, Switzerland.

**Results:**

The simulated and real-world driving studies involved 9 and 10 participants with type 1 diabetes, respectively. Both studies showed significant results in self-reported emotional reactions (*P*<.001). In simulated driving, neither warning modality nor blood glucose phase significantly affected self-reported arousal, but in real-world driving, both did (*F*_2,68_=4.3; *P*<.05 and *F*_2,76_=4.1; *P*=.03). Warning modality affected self-reported valence in simulated driving (*F*_2,68_=3.9; *P*<.05), while blood glucose phase affected it in real-world driving (*F*_2,76_=9.3; *P*<.001). Skin conductance response did not yield significant results neither in the simulated driving study (modality: *F*_2,68_=2.46; *P*=.09, blood glucose phase: *F*_2,68_=0.3; *P*=.74), nor in the real-world driving study (modality: *F*_2,76_=0.8; *P*=.47, blood glucose phase: *F*_2,76_=0.7; *P*=.5). In both simulated and real-world driving studies, the voice+LED warning modality was the most effective (simulated: mean 3.38, SD 1.06 and real-world: mean 3.5, SD 0.71) and urgent (simulated: mean 3.12, SD 0.64 and real-world: mean 3.6, SD 0.52). Annoyance varied across settings. The standard warning modality was the least effective (simulated: mean 2.25, SD 1.16 and real-world: mean 3.3, SD 1.06) and urgent (simulated: mean 1.88, SD 1.55 and real-world: mean 2.6, SD 1.26) and the most annoying (simulated: mean 2.25, SD 1.16 and real-world: mean 1.7, SD 0.95). In terms of preference, the voice warning modality outperformed the standard warning modality. In simulated driving, the voice+LED warning modality (mean rank 1.5, SD rank 0.82) was preferred over the voice (mean rank 2.2, SD rank 0.6) and standard (mean rank 2.4, SD rank 0.81) warning modalities, while in real-world driving, the voice+LED and voice warning modalities were equally preferred (mean rank 1.8, SD rank 0.79) to the standard warning modality (mean rank 2.4, SD rank 0.84).

**Conclusions:**

Despite the mixed results, this paper highlights the potential of implementing voice assistant–based health warnings in cars and advocates for multimodal alerts to enhance hypoglycemia management while driving.

**Trial Registration:**

ClinicalTrials.gov NCT05183191; https://classic.clinicaltrials.gov/ct2/show/NCT05183191, ClinicalTrials.gov NCT05308095; https://classic.clinicaltrials.gov/ct2/show/NCT05308095

## Introduction

### Overview

Around 9 million adults worldwide experience type 1 diabetes mellitus (T1DM) [1]. One of the most relevant acute complications associated with T1DM is hypoglycemia (ie, low blood glucose). This condition is associated with impaired cognitive, executive, and psychomotor function [2-4] and is linked to driving mishaps [5-7].

Previous work introduced the development of a voice warning for hypoglycemia while behind the wheel, whereas the voice assistant (VA) would work as a warning interface [8]. The hypoglycemia warning was intended as an app compatible with the VA that is already available in the car and that would allow delivering an alert in a hands-free manner. The study reported on the iterative development and evaluation of an in-vehicle hypoglycemia voice warning. It demonstrated that it is deemed useful and effective by drivers with T1DM, especially if the warning is kept simple and direct (ie, avoiding initiating a conversation with the driver). However, the paper did not investigate the effect of proactive behavior in the VA in such a context. Proactivity in VAs can cause a startling reaction, which is prone to annoyance [9] and driving impairments [10-12].

Ambient lighting can communicate with drivers without distracting them from their main task [13] or eliciting a strong emotional response [14]. This interface has been investigated as an indicator of several driving-relevant events, such as obstacle warnings or vehicle-state communication [15]. However, to the best of our knowledge, in-vehicle ambient lighting through LEDs has never been investigated as an indicator of a critical health state.

Our previous work [8] tested the concept of an in-vehicle voice warning delivered by the VA with healthy participants and then with individuals with T1DM, both in a driving simulator and a real car. The concept was developed following an iterative approach, and study participants provided feedback that we used to enhance the voice warning and test it on new participants. Thus, our previous work focused on technology acceptance and on improving it through user feedback. However, the voice warning was not evaluated against a standard warning (ie, beep with text), which could be used as a benchmark. Moreover, our previous work did not focus on the emotional reaction generated by getting a warning while driving. Therefore, this work investigates the effect of LEDs (ie, a possible solution to alleviate emotional reaction) and is to be understood as a continuation of our previous work. To foster experimental control and external validity, the same procedure is replicated in a simulated driving setting (ie, a computer simulator) and a real-world driving setting (ie, a car in a closed circuit).

### Background

#### Hypoglycemia Warnings

Hypoglycemia is a common complication of diabetes. The monitoring of blood glucose is essential to prevent hypoglycemia. Intermittent self-monitoring of blood glucose, flash glucose monitoring, and continuous glucose monitoring are commonly used methods. However, these methods are not adapted to the in-vehicle context, as they require the driver to visually attend to a handheld mobile device displaying the current blood glucose value. This behavior is known to impair driving performance [16], thus leading to dangerous situations while driving.

Tentative hands-free solutions have been proposed to address this issue in academia [17] and in the community of individuals with T1DM [18]. Specifically, prior research [17] suggested using vehicles as a platform to display blood glucose data on infotainment screens. Moreover, a digital community [18] created an open-source program to show their continuous glucose monitoring data on infotainment screens. However, these solutions are limited to visual information display, thus failing to be ergonomically suitable for the in-vehicle context while driving. Therefore, solutions must be developed, which can provide hypoglycemia warnings while driving. One approach is to use voice-first warnings (ie, delivered by the built-in in-vehicle VA), where the driver can be informed of the issue without having to attend to a display.

#### In-Vehicle Warnings

In-vehicle health-state warning systems are a part of advanced driver–assistance systems [19]. From a human-computer interaction perspective, in-vehicle warnings should be effective and communicate urgency without being annoying [20]. Currently, in-vehicle warnings vary from classic car warnings, visually presented on the dashboard with traffic-light colors and unspecific tones, to advanced driver–assistance system warnings that use visual, auditory, and haptic modalities [21]. Even though the visual signal should be redundant to the auditory and haptic signals, some driver-state warnings, such as the driver attention alert, are predominantly visual (eg, mug symbol with an indicative text such as “Time for a break”).

To decrease the demand for drivers’ visual attention, it is necessary to develop attention-attractive warnings without relying on visual displays as the main source of information. One approach would be to use the in-vehicle VA already built into the car to warn or alert drivers for critical situations, such as hypoglycemia (or drowsiness). This approach could ensure warnings’ effectiveness while reducing drivers’ visual distraction.

#### In-Vehicle VAs

VAs are increasingly being integrated into cars [22-24], allowing digital health interventions to be delivered via such an interface in a scalable way. In the in-vehicle context, VAs have ergonomic and experiential advantages: they reduce visual distraction compared to other infotainment technologies [25], foster a natural interaction [26], create a sense of social presence [27], and increase engagement [28]. Ultimately, VAs can create a sense of being in the presence of a copilot [29]. Therefore, in-vehicle VAs have great potential for delivering real-time and effective hypoglycemia warnings to drivers while driving.

#### Proactive VAs and the Risk of Startle

Proactivity is not part of the current common mental model of a VA. However, it does not necessarily affect driving performance [30] and is well-accepted by drivers [31,32]. Nevertheless, a sudden auditory stimulus can create a startling reaction, which could interfere with driving performance [10,33]. Hence, when it comes to critical situations such as hypoglycemia [34], it is important to develop warnings that gradually prepare the driver to be receptive to them. Ambient lighting can be used to gradually prepare the driver and add information without consequentially distracting the driver [13]. This technology has been previously investigated for in-vehicle driving behavior support such as collision and blind warnings, lane change decision support, and speed and attention direction recommendations [15]. In addition, it has been investigated to inform the driver about the vehicle’s decision-making in autonomous cars [35].

### Objectives

To this end, a hypoglycemia warning delivered by a mock-up of a built-in in-vehicle VA is designed and tested with individuals with T1DM and compared to a standard format of in-car warning (ie, unspecific alert tone with visual information) regarding driver experience.

Specifically, this work aims (1) to design a hands-free multimodal health intervention for hypoglycemia (ie, warning) compatible with the in-vehicle context and (2) to investigate the effect of warning modality (visual, vocal, and vocal with ambient lighting) on the emotional reaction and the acceptance of such technology.

## Methods

### Overview

Two studies were carried out, a simulated driving study and a real-world driving study. Across these studies, participant recruitment, study design, material and apparatus, procedure, and data analysis were the same. The difference lied in the setting. For this reason, all the following subsections, except for *Setting*, are described only once.

### Participants

#### Sampling, Inclusion Criteria, and Compensation

Patients diagnosed with T1DM attending the diabetes outpatient clinic of the Bern University Hospital were recruited. A physician (VFL) of the study team performed recruitment during regular outpatient visits with a face-to-face assessment. For the simulated driving study, participants were recruited between November 2021 and March 2022. For the real-world driving study, participants were recruited between April and June 2022. Inclusion criteria were age between 21 and 60 years, hemoglobin A_1c_≤9% (ie, a blood test indicating how well the patient’s diabetes is being controlled), functional insulin treatment (with insulin pump therapy or multiple daily injections) for at least 3 months with good knowledge of insulin self-management, possession of a Swiss driver’s license at least 3 years before study inclusion, and have driven at least once in the last 6 months. Each participant received an expense allowance of US $209.62 to cover general expenses caused by study participation (eg, transport).

#### Experience and Beliefs Questionnaires

Upon inclusion in the study, participants were asked to report the frequency of driving per week, their previous use of in-vehicle VAs, and their technology affinity. Technology affinity was assessed with the 16-item Technology Readiness Index [36]. This scale measures constructs susceptible to influencing the adoption of cutting-edge technology, such as optimism, innovativeness, discomfort, and insecurity.

### Study Design

The study was designed as quasi-experimental with 2 independent variables, that is, blood glucose phase and warning modality, and 1 main dependent variable, that is, emotional reaction. The blood glucose variable had 3 levels, that is, euglycemia, decreasing, and hypoglycemia (see the *Procedure* section). The warning modality variable had 3 levels as well, that is, standard, voice, and voice+LED (see the *Warning* section). The blood glucose phase was varied in a nonrandomized fashion (see the *Procedure* section), while the warning modality was pseudorandomized, and each modality was crossed with each blood glucose phase. Secondary outcomes included self-reported user experience measures, such as warning acceptance, perceived urgency, alerting effectiveness, annoyance, and preference.

### Material and Apparatus

#### Overview

In this section, the operationalization of the design variables is described. An overview is listed in Table 1.

**Table 1:** Study design variables.

Variable	Tool	Levels or values
Blood glucose^a^	Controlled hypoglycemia protocol [37]	Normal, decreasing, and hypoglycemia
Warning modality^a^	Hypoglycemia warning app [38]	Standard, voice, and voice+LED
Emotional reaction (objective)^b^	Empatica E4	Skin conductance response
Emotional reaction (subjective)^b^	Affective Slider [39]	Score (0-100)
Warning perceived urgency^b^	Baldwin and Moore scale [20]	Score (1=very urgent and 5=very insignificant)
Warning alerting effectiveness^b^	Baldwin and Moore scale [20]	Score (1=effective and 5=ineffective)
Warning annoyance^b^	Baldwin and Moore scale [20]	Score (1=I dislike it very much and 5=I like it very much)
Warning acceptance^b^	Van Der Laan Acceptance Scale [40]	Score (–2=negative extreme and +2=positive extreme)
Warning preference^b^	Arbitrary 3-point scale	Rank (1=best and 3=worst)

^a^Manipulation.

^b^Measure.

#### Blood Glucose

Blood glucose was manipulated by inserting 2 intravenous catheters: one for blood glucose measurement with an interval of 5-10 minutes and the other for the infusion of a combination of insulin and glucose, according to the patient’s current blood glucose and the experimental target blood glucose range. Euglycemia (ie, normal blood glucose) was defined as a concentration of 5-8 mmol/L; decreasing blood glucose was identified when blood glucose was below the euglycemia range (5-8 mmol/L) and progressing toward a target hypoglycemic range (3-3.5 mmol/L); and hypoglycemia was defined as a concentration of 3-3.5 mmol/L. For more technical details, refer to related research [37].

#### Warning

The Hypoglycemia warning app operationalized into a tablet (model SM-T590, Samsung) simulated the infotainment system’s touchscreen, and LED strips (RGB Light Strip Pro, Cololight) simulated the interior ambient lighting. The warning system was controlled through the Wizard of Oz method [41], that is, the system was controlled by the experimenter behind the scene and acting as if it was fully automated.

The LED strips had 60 LEDs per meter and could be remotely controlled with the Cololight app (Klaus Stephan GmbH). The tablet was used to run the Hypoglycemia warning app (publicly available on GitHub [38]), remotely controlled via a remote desktop app (AnyDesk, AnyDesk Software GmbH).

**Figure 1:**
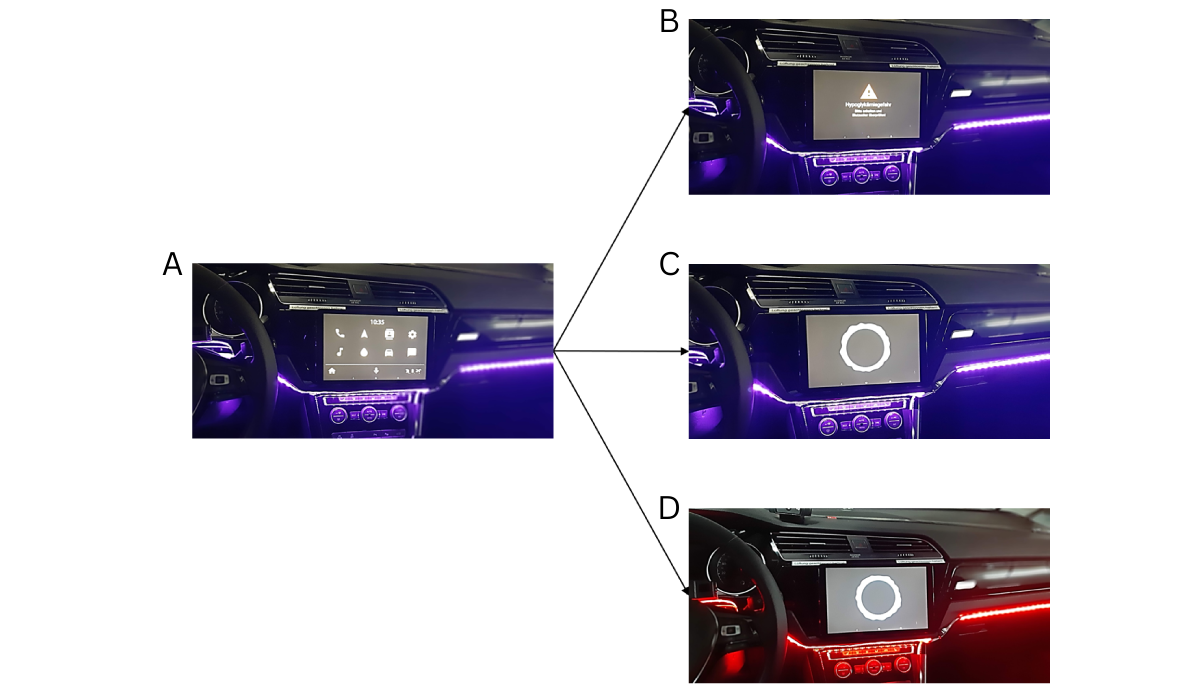
Illustration of the warning app and modalities. (A) The default state was shown during the drive and simulated the infotainment menu of the car. The (B) standard, (C) voice, and (D) voice+LED modalities were activated when a warning was delivered.

The warning system had 4 possible states: a default state and 3 intervention modalities (standard, voice, and voice+LED). The default state involved the LED strip being turned on in blue and the tablet showing a fake navigation menu (Figure 1A). The standard modality displayed a yellow warning sign with an informative text and was accompanied by an earcon. The text said “Risk of hypoglycemia. Please pull over and verify blood sugar.” The LED strips remained blue (Figure 1B). The voice modality displayed a VA animation accompanied by a prerecorded synthesized female voice (de-DE-Wavenet-C with speed=0.85 and pitch=–3.20, Google Inc). The voice said “I have detected a risk of hypoglycemia. Please pull over safely and verify your blood sugar” (translation from the German formulation “*Ich habe eine Hypogefahr erkannt. Bitte sicher anhalten und deinen Blutzucker überprüfen*”). The voice warning was designed based on the results reported in our previous work [8]. Once again, the LED strips remained blue (Figure 1C). The voice+LED modality displayed the same VA animation and prerecorded synthesized female voice but, before the onset of the voice warning, the LED strips turned red (Figure 1D).

Participants knew that they would receive a warning during each drive. Still, the warning presentation was pseudorandomized, where they would receive a complete permutation of the 3 warning modalities within a blood glucose phase. Participants were not explicitly informed about which warning was “the intervention of interest” and which one was the “comparator.”

#### Objective Emotional Reaction

Emotional reaction was measured physiologically through skin conductance response (SCR). SCR is the result of the sympathetic nervous system promptly regulating the activity of the sweat glands in response to a stimulus. This measure is associated with emotional arousal [42] and can be used to measure event-related emotional reactions objectively [43]. In this study, the Empatica E4 (Empatica Inc), a Conformité Européenne–certified wristband collecting physiological data in real time, was used. Participants wore the E4 during the main visit (see the *Procedure* section). Note that this measure provided solely the arousal dimension of emotion.

#### Subjective Emotional Reaction

Emotional reaction was also measured subjectively through the Affective Slider [39]. This digital scale is a self-reporting tool measuring valence and arousal on 2 separate sliders. Participants did not see any numerical anchor, but the score ranged from 0 to 100. Thus, valence is rated between a frowning and a smiling face (0=frowning and 100=smiling), and arousal between a sleepy and a widely awake face (0=sleepy and 100=widely awake).

#### Warning Perceived Urgency, Alerting Effectiveness, and Annoyance

To measure the perceived urgency mapping of the 3 modalities, for each modality, participants rated the perceived urgency, alerting effectiveness, and annoyance according to a scale from prior work [20]. The 3 dimensions were rated using a 5-point Likert scale (1=very urgent or effective or I like it very much and 5=very insignificant or ineffective or I dislike it very much). This questionnaire was filled out during the posttest visit (see the *Procedure* section).

#### Warning Acceptance

To compare the acceptance of the 3 modalities, participants filled out the Van Der Laan Acceptance Scale [40], once per modality. This scale consists of the 2 constructs, usefulness and satisfaction, with items answered on a 5-point semantic differential from –2 to +2, which means participants had to select a point between 2 opposite adjectives (eg, unpleasant or pleasant). This questionnaire was filled out during the posttest visit (see the *Procedure* section).

#### Warning Preference

To formalize their preference, patients were asked to rank the 3 modalities from best to worst. The scale was implemented as a radio button questionnaire with 1 item per modality (ie, beep with a warning sign and text, voice, and LED with voice) and a 3-point scale (ie, 1=best and 3=worst). Participants were also encouraged to provide comments to their answers, which were topically (ie, without verbatim transcription) recorded in written form. This questionnaire was filled out during the posttest visit (see the *Procedure* section).

### Setting

#### Simulated Driving

**Figure 2:**
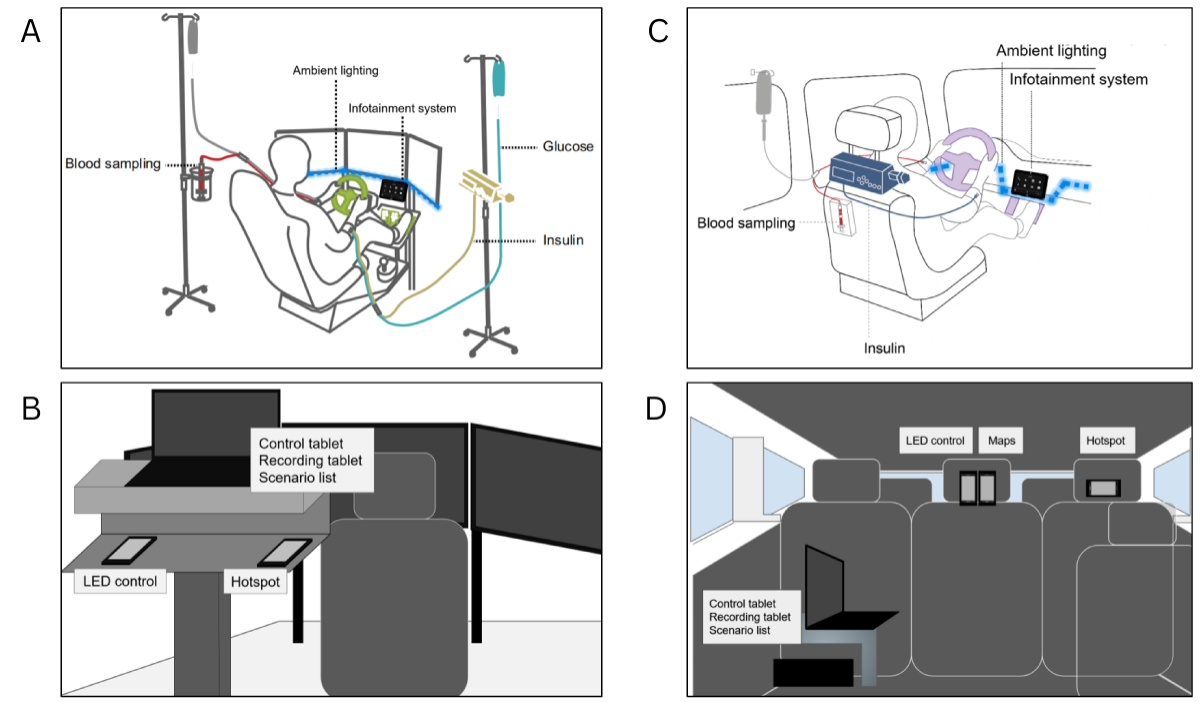
Comparison of patient’s and experimenter’s setup between simulated and real-world driving. The left figures represent the simulated driving setting, the right figures represent the real-world driving setting, the top figures (A and C) show the patient’s setup, and the bottom figures (B and D) show the experimenter’s setup.

Patients used a driving simulator (Carnetsoft Inc), featuring 3 monitors displaying the front, left, and right views of the driving cabin. The central monitor also displayed the cockpit and navigation arrows, which directed the patient through the environment. To control the simulator, participants used a steering wheel and pedals (Logitech Driving Force G29, Logitech), set to the automatic transmission. The simulator was connected to a stereo speaker and maintained at a constant volume (Figure 2A and C). The infotainment system simulator tablet (see the *Warning* section) was placed under the right side of the central monitor and connected to the simulator’s sound system. The LED lights were attached at the bottom of the 3 monitors. Figure 2A and 2C illustrates the patient’s setup.

The experimenter was standing behind the patient and controlled the LED stripes with a smartphone (Redmi Note, Xiaomi Inc) and the tablet via a laptop computer (ThinkPad X1 Carbon, Lenovo PC HK Ltd). A stopwatch app was used to manually onset the warning. Figure 2B and 2D illustrates the experimenter’s setup.

Three environments were used, namely, highway, countryside, and town, with the highway being the easiest to navigate due to variable traffic but no turns, the countryside having a moderated amount of traffic and turns, and the town being the most difficult with the most turns and traffic. Participants drove in the environments for about 5 minutes before receiving a hypoglycemia warning (run-in phase).

#### Real-World Driving

Patients drove in a minivan (Touran, Volkswagen) with an automatic transmission. The car was equipped with dual pedals to allow for intervention from a trained driving instructor, in case of emergency. In case the instructor needed to intervene, the event was recorded.

The infotainment system simulator tablet (see the *Warning* section) was placed on top of the infotainment screen and connected to the car’s sound system, maintained at a constant volume. The LED lights were attached along the cockpit from the left to the right extremities, passing by under the steering wheel, the infotainment system, and above the aperture of the glove compartment. The experimenter was sitting in the third row of the car and controlled the LED stripes with a smartphone and the tablet via a laptop computer (6th Gen ThinkPad X1 Carbon, Lenovo PC HK Ltd). A Google Map was used to manually onset the warning.

Patients were exposed to real-world driving on a test track provided by the Swiss Federal Department of Defense, Civil Protection and Sports. The driving scenarios on the track were designed to correspond with simulated environments used in the simulator setting (ie, highway, countryside, and town), featuring various driving elements such as turns, crossroads, stop signs, and a pedestrian crossing equipped with a dummy. As traffic simulation was not feasible, artificial obstacles, including boxes and traffic pylons, were used. Participants drove in the environments for 5-7 minutes before receiving a hypoglycemia warning (run-in phase).

### Ethical Considerations

The experiments were approved within the context of this project by the cantonal ethics commission of Bern, Switzerland (BASEC2020-00685 and BASEC2021-02381). Before any study-related procedure, informed consent specifying the analysis and the study protocol presented in this paper was obtained in written form from all participants. All collected data were deidentified by associating individual data to a numerical identification number. The data reported in this paper are part of the HEADWIND Study, a clinical trial registered under ClinicalTrials.gov (Part 3: NCT05183191 and Part 4: NCT05308095).

### Procedure

The procedure was divided into 3 visits. In a pretest visit, patients were welcomed to the Bern University Hospital, informed about the procedure and the warnings, and asked to fill out demographic and experience and beliefs questionnaires.

In the main visit, participants were welcomed to the relevant setting for the blood glucose manipulation and the objective emotional reaction measurements. Participants were aware that their blood sugar would be manipulated to reach specific goal ranges, but they were not informed of their blood glucose during the experiment. In the real-world driving setting, the driving instructor was also aware of the blood glucose manipulation and was blinded to the current blood glucose. The experimental team was not blinded to the blood glucose level.

**Figure 3:**
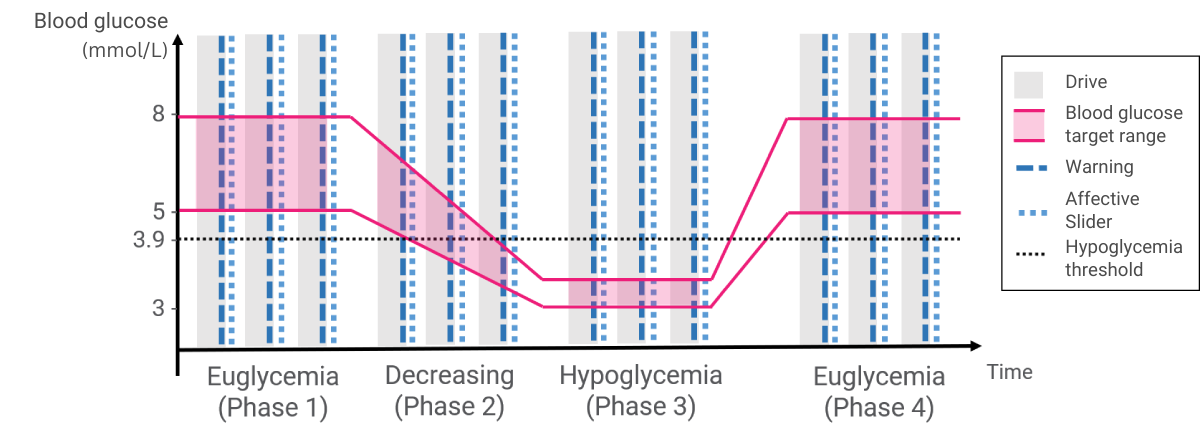
Overview of the procedure. The vertical gray bars represent the drives, the ribbon delimited by solid lines represents the blood glucose manipulation, the vertical dashed lines represent the warning deliveries, the vertical dotted lines represent the Affective Slider submission, and the horizontal dotted line represents the hypoglycemia threshold.

Each participant went through a fixed sequence of blood glucose phases: (1) a first drive with normal blood glucose (phase 1: euglycemia), where participants were first experiencing all types of environment and warning (and was thus considered as training); (2) a phase where blood glucose was progressively decreasing toward the moderate hypoglycemia threshold (ie, <3.0 mmol/L) with a target range of 3-3.5 mmol/L (phase 2: decreasing); (3) a phase with stable moderate hypoglycemia (phase 3: hypoglycemia); and (4) a final phase with normal blood glucose (phase 4: euglycemia). A warning was delivered at the end of each drive to explore the effect of the blood glucose phase. Participants drove in the 3 types of environments in each phase. The sequence of environment type was pseudorandomized [8], that is, participants were exposed to all 3 warning modalities within each phase, but the sequence of modalities within 1 phase was random. Similarly, the warning modality was pseudorandomized to balance modality with environments. Once participants received a warning, they were expected to stop, and the drive came to an end. At the end of each drive, participants filled out the Affective Slider referring to the warning they just received. Figure 3 shows an overview of the procedure.

In a posttest visit, participants were once more exposed to the 3 warning modalities and were required, after each exposure, to fill the Baldwin and Moore scale [20] and the Van Der Laan Acceptance Scale. Finally, they ranked the 3 warning modalities.

### Data Analysis

The continuous variables of sample characteristics (ie, demographics and previous experience) are presented with mean and SD. Frequency variables of sample characteristics are presented in count numbers (ie, n) and percentages of the total experiment sample.

Emotional reaction (objective and subjective) measures were analyzed as a function of blood glucose (excluding phase 1) and warning modality and verified with a mixed-effects linear model, ANOVA test, and a significance threshold of *P*=.05. Effect size was calculated with partial η^2^ (0.01 indicates a small effect, 0.06 indicates a medium effect, and 0.14 indicates a large effect). Moreover, the objective emotional reaction was analyzed following established guidelines [43]: SCR (ie, rapid phasic component) was standardized for individual differences by dividing the SCR signal by the individual maximum SCR and by reducing the noise. In addition, SCR was calculated by considering the change in skin conductance between the average skin conductance in the 5-second window before the warning onset and the average skin conductance in the 5-second window after the warning onset itself (including latency of 1 second). For each measure of emotional reaction, a mixed-effects linear model was estimated with warning modality (3 levels: standard, voice, and voice+LED) and blood glucose (3 levels: normal, decreasing, and hypoglycemia) as independent variables and with emotional reaction measure (ie, either self-reported arousal, self-reported valence, or SCR) as the dependent variable.

Warning evaluations (ie, perceived urgency, alerting effectiveness, annoyance, and acceptance) were aggregated with means and SDs, presented in a table. Moreover, perceived urgency, alerting effectiveness, and annoyance were centered on 2 to match the acceptance scores, for the sake of comparison.

Preference ranking was aggregated across modalities in the frequency of rank as best, middle, or worst (ie, how many times 1 modality was ranked as the best, middle, or worst in comparison to the other 2). If participants were commenting on their choices, highlight note-taking was performed.

Data analysis and graphical representations were performed using RStudio (Posit Software) packages such as *lmerTest* or mixed-effects linear modeling and *ggplot2* and patchwork for data visualization. All results are separated by experiment (ie, simulated vs real-world driving) and juxtaposed to allow direct comparison.

## Results

### Overview

The data of 2 participants of the simulated driving study were excluded due to partial data loss. In the real-world driving setting, the driving instruction had to intervene in 1 instance, as the participants did not follow the driving path (ie, did not turn left) during phase 4 (ie, while in euglycemia).

### Sample Characteristics

Overall, the majority were male participants, who drove multiple times per week and did not have previous experience with in-vehicle VAs. Participants were approximately 40 years of age and had a Technology Readiness Index between 3.5 and 4 (over a maximum of 5). Details are shown in Table 2.

**Table 2:** Sample characteristics across studies.

Characteristics		Simulated (n=9)	Real-world (n=10)
**Sex, n (%)**			
	Male	8 (89)	7 (67)
	Female	1 (11)	3 (33)
Age (years), mean (SD)		45.7 (11.79)	37.3 (11.1)
**Frequency of driving, n (%)**			
	1 time per month	1 (11)	1 (10)
	2-5 times per month	—^a^	2 (20)
	2-5 times per week	5 (56)	4 (40)
	Every day	3 (33)	3 (30)
**Previous use of in-vehicle voice assistants, n (%)**			
	Never	4 (44)	6 (60)
	Rarely	1 (11)	2 (20)
	Sometimes	2 (22)	1 (10)
	Often	2 (22)	1 (10)
TRI^b^ (over a maximum of 5), mean (SD)		3.6 (0.62)	3.9 (0.5)

^a^Not available.

^b^TRI: Technology Readiness Index.

### Emotional Reaction

#### Overview

In this section, the results of the self-reported and physiological measures of emotional reaction (ie, self-reported valence and arousal and SCR) are described. A mixed-effect model was run on all these measures, with warning modality and blood glucose phase as independent variables.

#### Self-Reported Arousal and Valence

**Figure 4:**
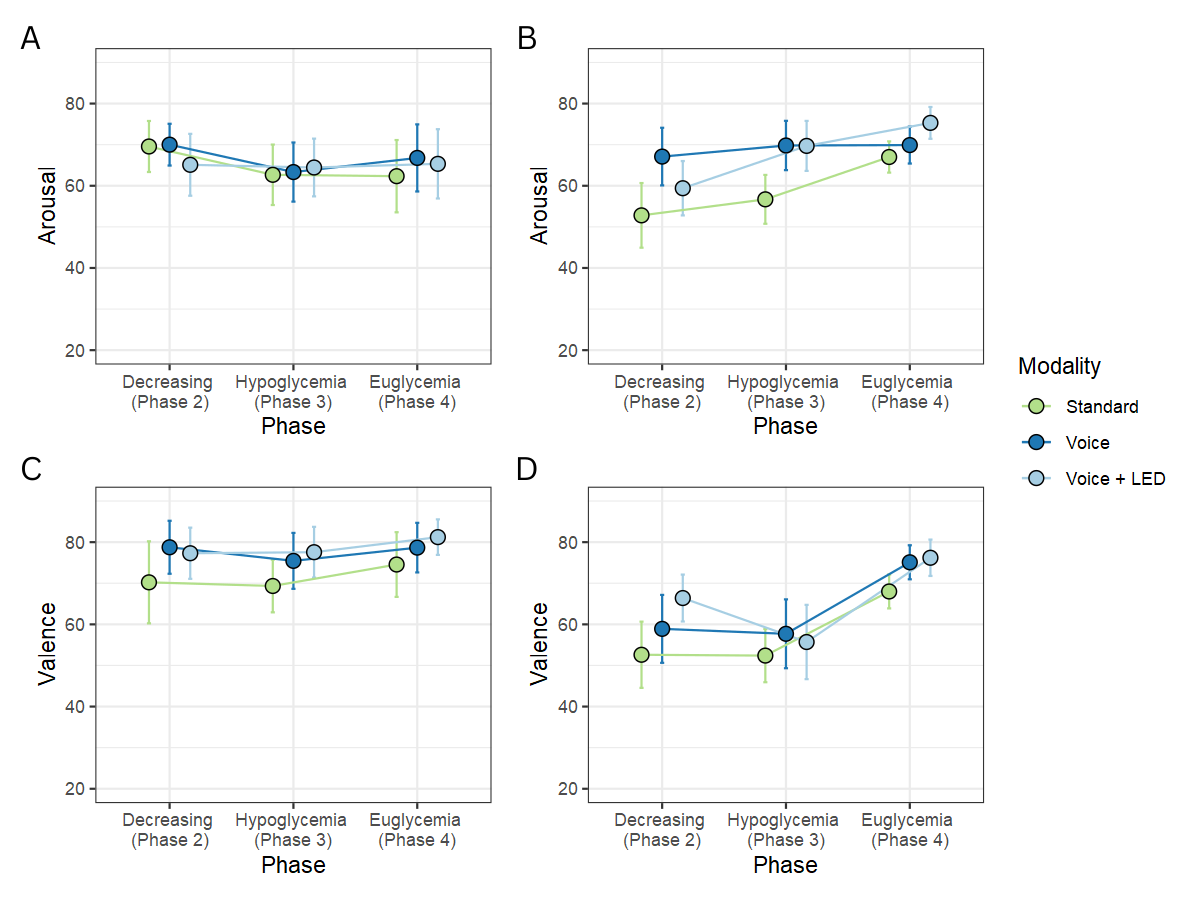
Line plots of arousal and valence across warning modality and blood glucose phases across (A and C) simulated and (B and D) real-world driving. Error bars represent SEs.

According to our results, the mixed-effect models were significant for both valence and arousal in both studies (*P*<.001). Figure 4 shows the means and SEs for arousal and valence.

In the simulated driving study, arousal was not significantly affected by either of the independent variables (modality: *F*_2,68_=0.1; *P*=.91; partial η^2^=0, blood glucose phase: *F*_2,68_=1.1; *P*=.35; partial η^2^=0.03). Valence was significantly affected by warning modality (*F*_2,68_=3.9; *P*=.03; partial η^2^=0.10) but not by blood glucose phase (*F*_2,68_=1.1; *P*=.35; partial η^2^=0.03).

In the real-world driving study, arousal was significantly affected both by warning modality (*F*_2,76_=4.3; *P*=.02; partial η^2^=0.10) and blood glucose phase (*F*_2,76_=4.1; *P*=.02; partial η^2^=0.10). Valence was significantly affected by blood glucose phase (*F*_2,76_=9.3; *P*<.001; partial η^2^=0.20) but not by warning modality (*F*_2,76_=2; *P*=.14; partial η^2^=0.05).

#### Physiological Arousal

According to our results, the mixed-effect models were not significant in both studies. Hence, the warning modality and blood glucose phase did not significantly affect physiological arousal measured via SCR neither in the simulated driving study (modality: *F*_2,68_=2.46; *P*=.09; partial η^2^=0.1, blood glucose phase: *F*_2,68_=0.3; *P*=.74; partial η^2^=0). nor in the real-world driving study (modality: *F*_2,76_=0.8; *P*=.47; partial η^2^=0, blood glucose phase: *F*_2,76_=0.7; *P*=.5; partial η^2^=0).

### Technology Acceptance

In this section, the technology acceptance results (ie, Baldwin and Moore scales of urgency, effectiveness, and annoyance and the Van Der Laan Acceptance Scale) are described. Details are available in Table 3 [44].

**Table 3:** Technology acceptance measure across studies.

Measure and warning modalities		Simulated driving, mean (SD)	Real-world driving, mean (SD)
**Urgency**			
	Standard	1.88 (1.55)	2.6 (1.26)
	Voice	2.88 (1)	3 (0.82)
	Voice+LED	3.12 (0.64)	3.6 (0.52)
**Effectiveness**			
	Standard	2.25 (1.16)	3.3 (1.06)
	Voice	2.75 (1.28)	3.6 (0.7)
	Voice+LED	3.38 (1.06)	3.5 (0.71)
**Annoyance**			
	Standard	2.25 (1.16)	1.7 (0.95)
	Voice	0.88 (0.84)	1 (0.82)
	Voice+LED	0.5 (0.76)	1.1 (1.2)
**Acceptance**			
	Standard	3.35 (0.55)	3.52 (0.53)
	Voice	3.74 (0.47)	3.88 (0.41)
	Voice+LED	3.86 (0.47)	3.77 (0.61)

In the simulated driving study, the voice+LED modality elicited the highest sense of urgency and effectiveness, the least annoyance, and the highest acceptance, followed by the voice modality. In real-world driving, the voice+LED modality elicited the highest sense of urgency and least annoyance, while the voice modality elicited the most sense of effectiveness and the highest acceptance.

### Preference Ranking

The average rank in the simulated driving study was 1.5 (SD 0.82) for the voice+LED modality, 2.2 (SD 0.6) for the voice modality, and 2.4 (SD 0.81) for the standard modality. The average rank in real-world driving was 1.8 (SD 0.79) for both the voice+LED and voice modalities and 2.4 (SD 0.84) for the standard modality.

In the real-world driving study, topical feedback showed that 6 participants mentioned that the light was not noticeable while driving (eg, “I have not noticed the light but at night, it certainly works better” [Participant 4]).

## Discussion

### Principal Findings

This study investigated the effect of warning modality (visual, vocal, and vocal with ambient lighting) on the emotional reaction and the acceptance of such a technology. Our results showed that voice warnings are more appreciated and considered more effective than standard warnings. However, the ambient lighting did affect such judgments.

### Effects of Warning Modality on Emotional Reaction

#### Effect on SCR

No significant effect of warning modality (or blood glucose phase) on skin conductance was found. SCR measured through Empatica E4 has been previously shown to be linked with response to stimuli [45-47]. Moreover, it has been associated with blood glucose variation [48,49]. Therefore, we may consider the possibility that the measurement protocol used in this research may have experienced certain weaknesses and have affected the validity of the obtained results. Hence, we cannot consider the lack of significant results as negative evidence. Nevertheless, future research should further investigate the startling effect of the voice warning while driving, either by replicating our experimental setting, by using alternative electrodermal activity measurement tools [50], or by using other measures of emotional reaction, such as eye blinks [44].

#### Self-Reports

Our results showed that although in simulated driving the effect of modality on self-reported arousal was not significant, this was the case in real-world driving. In particular, higher arousal was observed during decreasing glucose and hypoglycemia for the voice and voice+LED modalities. Moreover, in the simulated driving study, the results on self-reported valence show a significant effect of modality, with voice+LED and voice warnings eliciting higher valence than a standard warning, particularly during decreasing glucose and hypoglycemia. This was not the case in the real-world driving study.

Despite the mixed results, the warning modality had a significant effect in the critical moments, that is, when the participants were about to experience or already experiencing hypoglycemia. Thus, our results showed the relevance of measuring emotional reaction at different levels of blood glucose. While the Affective Slider is a very efficient measurement tool, it is important to note that some participants expressed a lack of confidence in self-reporting their emotions with it. Thus, future research might benefit from using alternative self-reported measures of emotion, such as the Positive and Negative Affect Schedule [51] or the Discreet Emotions Questionnaire [52].

#### Mixed Results

These mixed results preclude the formulation of definitive conclusions regarding the effect of modality on emotional reaction based on this study. As a sudden auditory stimulus can create a startling reaction, which can interfere with driving performance [10,33], future research should consider our recommendations and further investigate the design of a warning that is both effective and nonstartling.

### Effects of Warning Modality on Acceptance and Preference

#### Acceptance

Our results demonstrated that the voice+LED modality tended to be the most valued regarding acceptance and preference. However, in the real-world driving study, this advantage, compared to the voice modality, seemed to decrease compared to the simulated driving study. This change might be due to the setup, where the ambient lighting was more visible in the laboratory than outdoors. Therefore, the advantage of the ambient lighting might have decreased in the real-world driving study. Thus, from the results, it is clear that the voice warning had an advantage over the standard warning, while the addition of ambient lighting (ie, voice+LED modality) did not bring a substantial advantage.

#### Preference

Finally, when asking participants for a posttest ranking of the warning modalities, results showed that voice (ie, both voice and voice+LED modalities) had a constant advantage over a beep with text (ie, standard modality). However, in the simulated driving study, the voice+LED modality was ranked first more often than in the real-world driving study, leading to interpret these results similar to the technology acceptance results, that is, adding ambient lighting to a voice warning was not considered substantially advantageous. These results might be influenced by the perception of the lights in daylight conditions, which differed between the simulated and the real-world driving settings. Based on the topical feedback from the participants experiencing the real-world driving setting, it might be that the contrast between the exterior and interior luminance was too low. As we did not measure the contrast in luminance between the daylight and the LEDs, future research should further investigate the use of ambient lighting as a component of in-vehicle warnings with greater control on luminance [53].

### Implications

This paper involves implications both from health and automotive perceptive. First, our investigation represents a step forward in managing a real-life hazard associated with hypoglycemia. Our previous work [8] focused on designing an effective voice warning. This work compared it to a standard warning with an unspecified auditory signal with a text, and with an addition of ambient lighting, to make the voice warning less abrupt. Our results show an advantage for a voice warning (ie, spoken) over a tone with text but not for ambient lighting. While participants showed a higher preference for adding ambient lighting in the simulated driving study, the way the ambient lighting was set in the real-world driving study was not noticeable enough to replicate the results. Other work has investigated more attention-grabbing ambient lighting, such as blinking lights [54]. Future research should investigate if different typologies of ambient light patterns could affect emotional reaction and acceptance.

Second, our investigation aims to inspire in-vehicle technology designers to develop in-vehicle health warnings using the in-vehicle VA. While there are driver-state warnings, they predominantly alarm the driver with an unspecific beep and a text on the cockpit. Future research should investigate how using the in-vehicle VA to warn the driver about dizziness or lack of attention would be accepted by drivers. Moreover, providing health-related warnings while driving fits the concept of “health-conscious” cars [55,56]. Along the lines of our investigation, there have been some attempts to develop blood glucose monitoring interfaces for the car [17,18]. However, they primarily rely on visual displays and are ill-adapted to the context of driving.

Finally, this study assumed the delivery of a hypoglycemia warning in a car with an autonomy of level 0 (ie, no automation) or level 1 (ie, with driver assistance). As cars are becoming increasingly automated, a hypoglycemia warning should be compatible with cars with a higher level of autonomy. However, the warning designed in this work is compatible with higher levels of automation. For instance, during autonomous driving, the in-vehicle VA could alert the driver that hypoglycemia has been detected and trigger the car to autonomously stop. During manual driving, the in-vehicle VA could warn the driver and trigger the car to take over (switch from manual to autonomous driving) and pull over.

### Limitations and Future Research

Despite our best efforts, this investigation involved certain limitations. First, the sample size was rather small. Nevertheless, valuable insights on digital solutions can be provided with a small sample size [57-59]. Thus, although it does not allow drawing conclusions on the interaction of drivers with T1DM with in-vehicle hypoglycemia warnings, it motivates further research in this domain.

Second, emotional response to the warning was evaluated in different blood glucose states and a controlled setting (ie, simulator and closed circuit). However, the warning was not delivered when relevant (ie, only when the driver was actually undergoing hypoglycemia) or while the participant was driving on a public road unaware of the upcoming critical state. While our method allowed controlling for the blood glucose in the emotional response, it did not allow us to find the opportune moment for intervention delivery, that is, at what point of upcoming hypoglycemia is the warning most appropriate (both in terms of emotional reaction and acceptance). Future research should investigate the effectiveness of such an intervention in a more realistic context, where the driver does not expect to be warned and is actually about to undergo hypoglycemia while on a public road.

### Conclusions

This paper proposes the use of the in-vehicle VA and ambient lighting system to deliver a hypoglycemia warning, ensuring a hands-free alert. The investigation focused on the extent to which warning modality could affect emotional response and acceptance both in a simulated and real-world environment. Although further investigations are needed, our results suggest, together with our previous work [8], that implementing multimodal warnings can improve the management of hypoglycemia in cars and also emphasize the potential of in-vehicle VA for delivering health-related warnings.

## Abbreviations

SCR: skin conductance response
T1DM: type 1 diabetes mellitus
VA: voice assistant

## Data Availability

The data sets generated and analyzed during this study are available from the corresponding author on reasonable request.
